# Fish oil omega-3 polyunsaturated fatty acids attenuate oxidative stress-induced DNA damage in vascular endothelial cells

**DOI:** 10.1371/journal.pone.0187934

**Published:** 2017-11-09

**Authors:** Chiemi Sakai, Mari Ishida, Hideo Ohba, Hiromitsu Yamashita, Hitomi Uchida, Masao Yoshizumi, Takafumi Ishida

**Affiliations:** 1 Department of Cardiovascular Physiology and Medicine, Graduate School of Biomedical and Health Sciences, Hiroshima University, Hiroshima, Japan; 2 Department of Cardiovascular Medicine, Fukushima Medical University, Fukushima, Japan; Niigata Daigaku, JAPAN

## Abstract

**Objective:**

Omega-3 fatty acids, particularly eicosapentaenoic acid (EPA) and docosahexaenoic acid (DHA), likely prevent cardiovascular disease, however their mechanisms remain unclear. Recently, the role of DNA damage in atherogenesis has been receiving considerable attention. Here, we investigated the effects of EPA and DHA on DNA damage in vascular endothelial cells to clarify their antiatherogenic mechanisms.

**Methods and results:**

We determined the effect of EPA and DHA on H_2_O_2_-induced DNA damage response in human aortic endothelial cells. Immunofluorescence staining showed that γ-H2AX foci formation, a prominent marker of DNA damage, was significantly reduced in the cells treated with EPA and DHA (by 47% and 48%, respectively). H_2_O_2_-induced activation of ATM, a major kinase orchestrating DNA damage response, was significantly reduced with EPA and DHA treatment (by 31% and 33%, respectively). These results indicated EPA and DHA attenuated DNA damage independently of the DNA damage response. Thus the effects of EPA and DHA on a source of DNA damage were examined. EPA and DHA significantly reduced intracellular reactive oxygen species under both basal condition and H_2_O_2_ stimulation. In addition, the mRNA levels of antioxidant molecules, such as heme oxygenase-1, thioredoxin reductase 1, ferritin light chain, ferritin heavy chain and manganese superoxide dismutase, were significantly increased with EPA and DHA. Silencing nuclear factor erythroid 2-related factor 2 (NRF2) remarkably abrogated the increases in mRNA levels of antioxidant molecules and the decrease in intracellular reactive oxygen species. Furthermore, EPA and DHA significantly reduced H_2_O_2_-induced senescence-associated β-galactosidase activity in the cells (by 31% and 22%, respectively), which was revoked by *NRF2* silencing.

**Conclusions:**

Our results suggested that EPA and DHA attenuate oxidative stress-induced DNA damage in vascular endothelial cells through upregulation of NRF2-mediated antioxidant response. Therefore omega-3 fatty acids likely help prevent cardiovascular disease, at least in part, by their genome protective properties.

## Introduction

Since the epidemiological study of Greenland Eskimos in the 1970s revealed the correlation between the high intake of omega-3 polyunsaturated fatty acids (n-3 PUFAs) and the low incidence of cardiovascular disease (CVD), the broad range of beneficial properties of n-3 PUFAs have been reported, such as anti-atherogenic, anti-thrombogenic and blood pressure-lowering effects [1, 2]. Thus, it is recommended to take n-3 PUFAs, particularly eicosapentaenoic acid (EPA) and docosahexaenoic acid (DHA), as a prescribed drugs or supplements, or consume oily fish, which is rich in n-3 PUFAs, to prevent CVD or to treat hypertriglyceridemia [3–6].

EPA and DHA are described not only to decrease plasma triglyceride levels but also to have anti-inflammatory effects and to improve endothelial function, all of which mediate anti-atherogenic effects [7–10]. The anti-inflammatory mechanisms of n-3 PUFAs have been getting clarified in some degree. For instance, n-3 PUFA-derived lipid mediators have been recently described, namely resolvins, protectins and maresins, which function in the resolution of inflammation [11]. Also, G-protein-coupled receptor 120 has been newly identified as an n-3 PUFA receptor, which potently mediates anti-inflammatory and insulin-sensitizing effects in monocytes/macrophage and adipocytes [12]. However, the mechanisms by which n-3 PUFAs modulate endothelial function are yet to be elucidated.

Recently the roles of DNA damage and the DNA damage response in atherosclerosis have been receiving considerable attention. This was first recognized in patients with progeroid syndromes, such as Werner syndrome and Hutchinson-Gilford syndrome, presenting the early onset of atherosclerosis [13]. The former syndrome is caused by mutations in genes encoding a DNA repair protein, whereas DNA damage is accumulated as a result of defects in nuclear envelope in the latter. These facts suggest a strong link between DNA damage and atherosclerosis. We and other investigators have previously reported the accumulation of DNA damage in human atherosclerotic lesions [14, 15]. The evidence indicates that DNA damage is involved in the development of atherosclerotic plaques. Numerous agents may cause DNA damage, but reactive oxygen species (ROS) are the most frequent cause [16]. Thus reducing ROS-induced DNA damage may be crucial for prevention of atherosclerosis and related CVDs. In this study, we investigated effects of EPA and DHA on chromosomal DNA integrity in human vascular endothelial cells to further identify their anti-atherogenic properties and possible molecular pathways involved.

## Materials and methods

### Reagents

EPA and DHA were purchased from SIGMA-ALDRICH (St. Louis, MO). Fatty-acid free BSA was purchased from Calbiochem (La Jolla, CA). Endothelial Cell Basal Medium (EBM)-2 was purchased from Lonza (Walkersville, MD). Antibody suppliers were following; anti-histone H2AX (phospho Ser139) (γ-H2AX), Millipore (Billerica, MA); anti-ATM, Santa Cruz Biotechnology (Santa Cruz, CA); anti-ATM (phospho S1981) and anti-DNA-PKcs (phospho S2056), Abcam (cambridge, UK); anti-catalase, Calbiochem; anti-DNA-PKcs (LAB VISION Co., Fremont, CA). Chloromethyl-2’,7’-dichlorofluorescein deacetate (CM-H_2_DCFDA) was purchased from Invitrogen (Eugene, OR).

### Cell culture

Human aortic endothelial cells (HAECs) were purchased from KURABO (Osaka, Japan), and cultured according to the previously described method [14]. Cells at passage 5 to 10 were used.

### Fatty acid treatment

EPA and DHA stock solutions were prepared by dissolving each reagent in ethanol to obtain the concentration of 100 mM. To conjugate each reagents to BSA, the stock solutions were diluted 1000 fold with a growth medium containing 1% fatty-acid free BSA and incubated at 37°C for 30 min.

### Detection of DNA double-strand breaks

The cells were exposed to H_2_O_2_ (100 μM) for 15 min and incubated in fresh media for 30 min or 24 h to allow DNA damage to be repaired. DNA double-strand breaks were detected by immunofluorescent analysis using anti-γ-H2AX antibody as previously described [14]. Briefly, cells were fixed with 4% paraformaldehyde and permeabilized with Triton X-100. The cells were incubated with anti-γ-H2AX for 30 min at 37°C and then with Cy3-conjugated secondary antibody for 30 min at 37°C. Nuclei were stained with DAPI. Samples were observed under an Axioplan2 microscope (Zeiss, Thornwood, NY). To calculate the γ-H2AX foci number per cell, γ-H2AX foci in at least 100 cells were counted.

### Western blotting

Western blotting was performed as previously reported [17].

### RNA preparation and real-time RT-PCR analysis

Total RNA was isolated from cells using TRIzol (Invitrogen, Carlsbad, CA) according to the manufacturer’s protocol, except using Ethachinmate (NIPPON GENE, Toyama, Japan) to improve RNA precipitation. RNA (2.0 μg) was reverse-transcribed into cDNA with random primer using ReverTra Ace (TOYOBO) as described in the manufacturer’s protocol. Real-time RT-PCR analysis was performed using CFX96 real-time PCR system (Bio-Rad Laboratories, Hercules, CA) and THUNDERBIRD SYBR qPCR Mix (TOYOBO) to detect levels of the mRNAs for heme oxigenase 1 (*HO-1*), NADPH dehydrogenase quinone 1(*NQO1*), ferritin heavy chain (*FTH*), ferritin light chain (*FTL*), thioredoxin reductase 1 (*TXNRD1*), superoxide dismutase 2 (*SOD2*), catalase, peroxiredoxin 5 (*PRDX5*), nuclear factor erythroid derived 2 (*NRF2*), interleukin 6 (*IL-6*), monocyte chemoattractant molecule 1 (*MCP-1*), 18s ribosomal RNA. The sequences of primes used in this study were described in S1 Table.

### Detection of intracellular ROS

Intracellular ROS was measured by chloromethyl-2’,7’-dichlorofluorescein diacetate (CM-H_2_DCFDA) staining. Cells were rinsed twice with Hank’s balanced salt solution (HBSS) prior to adding CM-H_2_DCFDA (10 μM), and then incubated with the reagent for 30 min in dark at room temperature. During the incubation the cells were stimulated with H_2_O_2_ (100 μM) for 30 min, and then washed with cold HBSS. The fluorescence was detected using a fluorescence plate reader ARVO X3 (PerkinElmer, Waltham, MA) with excitation of 488 nm and emission of 530 nm.

### *NRF2* and *FOXO1* silencing

Cells were transfected with small interfering RNA (siRNA) against human *NRF2* (Hs_NFE2L2_7 or 9, QIAGEN, Valencia, CA) and/or human *FOXO1* (Hs_FOXO1A_4, QIAGEN), or negative control cocktail (siTrio Negative Control, B-Bridge International, Mountain View, CA) using DharmaFECT1 (Dharmacon Research, Lafayette, CO) according to the manufacturer’s protocol.

### H_2_O_2_-induced cell senescence

Cells were exposed to H_2_O_2_ (100 μM) for 1 h and then incubated for 5 days with medium being refreshed every other day. Cell senescence was detected by senescence-associated β-galactosidase (SA-β-gal) staining using a senescence cells staining kit (SIGMA-ALDRICH) according to the protocol specified by the manufacturer. To calculate the percentage of SA-β-gal positive cell, at least 500 cells were counted for each sample. Bright-field and fluorescent images were captured using a fluorescence microscope BZ-X700 (KEYENCE Co., Osaka, Japan). Average fluorescent intensity of each sample was analyzed.

### Statistical analysis

Data are expressed as mean ± SEM. Student’s *t*-test and Wilcoxon signed-rank test were used to determine statistical differences between the two groups. Statistical significance was established at a *P* value less than 0.05.

## Results

### EPA and DHA attenuate H_2_O_2_-induced DNA double-strand breaks in HAECs

**Fig 1:**
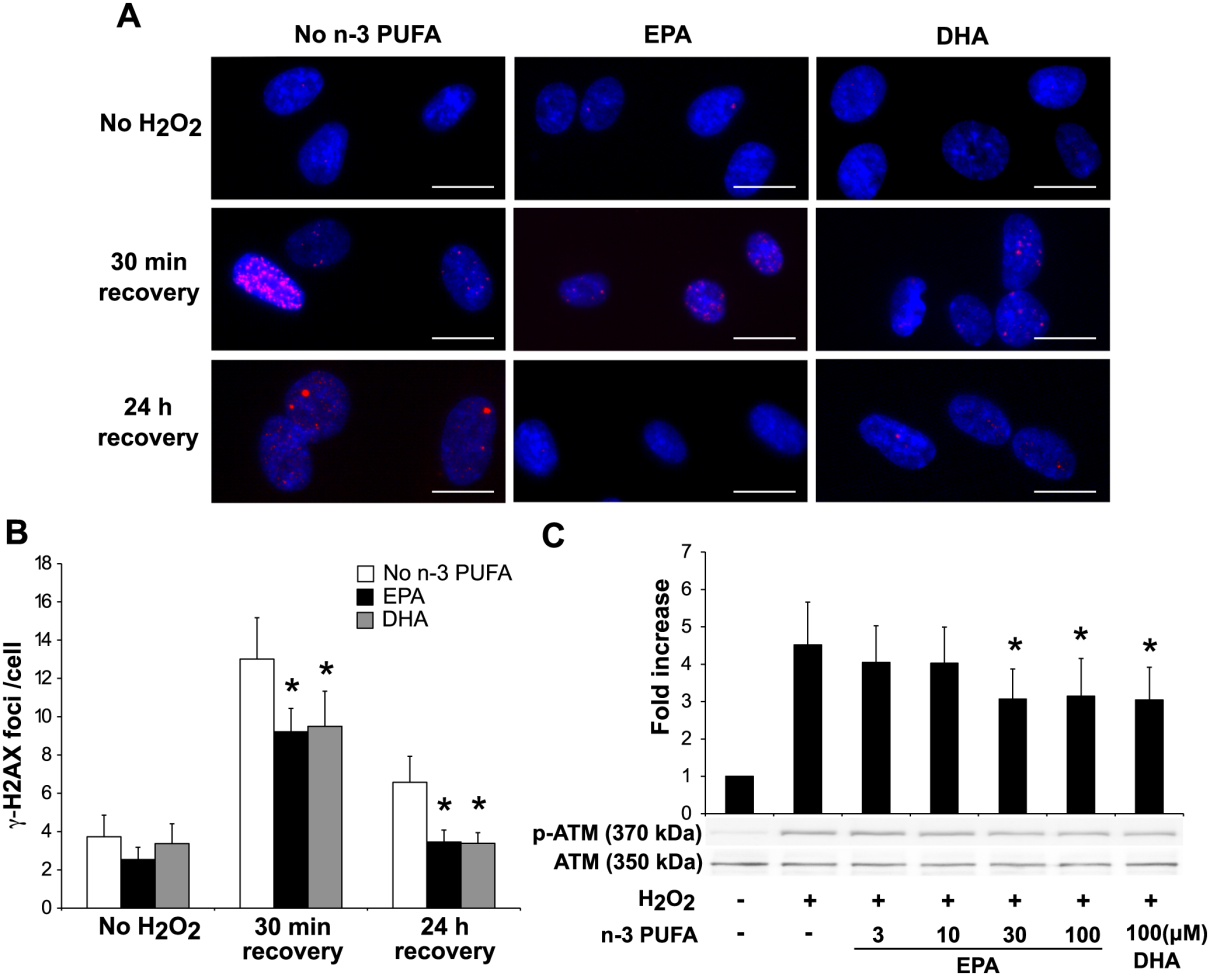
n-3 PUFAs attenuate H_2_O_2_-induced DNA damage and DNA damage response in HAECs. (A) Immunofluorescent staining of γ-H2AX (red) in HAECs. Cells were treated with EPA or DHA (100 μM) for 48 h prior to incubation with H_2_O_2_ (100 μM) for 15 min, and then changed to normal medium and incubated for 30 min or 24 h as a recovery period. Representative images of γ-H2AX foci are shown. Scale bar = 20 μm (B) Quantitation of γ-H2AX foci. γ-H2AX foci number was divided by the total cell number, which was expressed as γ-H2AX foci/cell. *P<0.05 compared with corresponding control (n = 4). (C) Western blot analysis of phosphorylated ATM (S1981). Cells were incubated with H_2_O_2_ (100 μM) for 1 h after 48 h treatment with EPA (indicated doses) or DHA (100 μM). Whole cell lysates were analyzed by immunoblotting. *P<0.05 compared with H_2_O_2_(+) control (n = 6).

We first examined whether EPA and DHA effect on H_2_O_2_-induced DNA damage in HAECs. Among various types of DNA damage, the most severe and difficult to repair is DNA double-strand breaks (DSBs) [18]. Thus we detected the phosphorylated form of the histone variant H2AX at serine 139 (γ-H2AX), which is the most prominent marker of DSBs [19]. Treatment with EPA and DHA significantly reduced γ-H2AX foci formation at 30 min (by 29.2% and 27.0%, respectively) and 24 h after H_2_O_2_ exposure (by 47.5% and 48.4%, respectively). At 24 h after H_2_O_2_-exposure, γ-H2AX foci formation of the cells treated with n-3 PUFAs was suppressed almost to the basal level while that of control remained increased by 76.4% (Fig 1A and 1B). We speculated that the decrease of DSBs by n-3 PUFA treatment was result of activating the DNA damage response, thereby DSBs being repaired. To test this, we examined the H_2_O_2_-induced activation of ATM, the major transducer of the DNA damage response [20]. Against our speculation, western blotting analysis showed that EPA (30 and 100 μM) and DHA (100 μM) significantly reduced phosphorylation of ATM (by 32.0%, 30.5% and 32.6%, respectively) (Fig 1C). We observed similar result for H_2_O_2_-induced activation of DNA-dependent protein kinase, an enzyme required for non-homologous end joining pathway of DNA repair (S1 Fig).

### EPA and DHA reduce intracellular oxidative stress in HAECs via upregulation of antioxidant molecules

**Fig 2:**
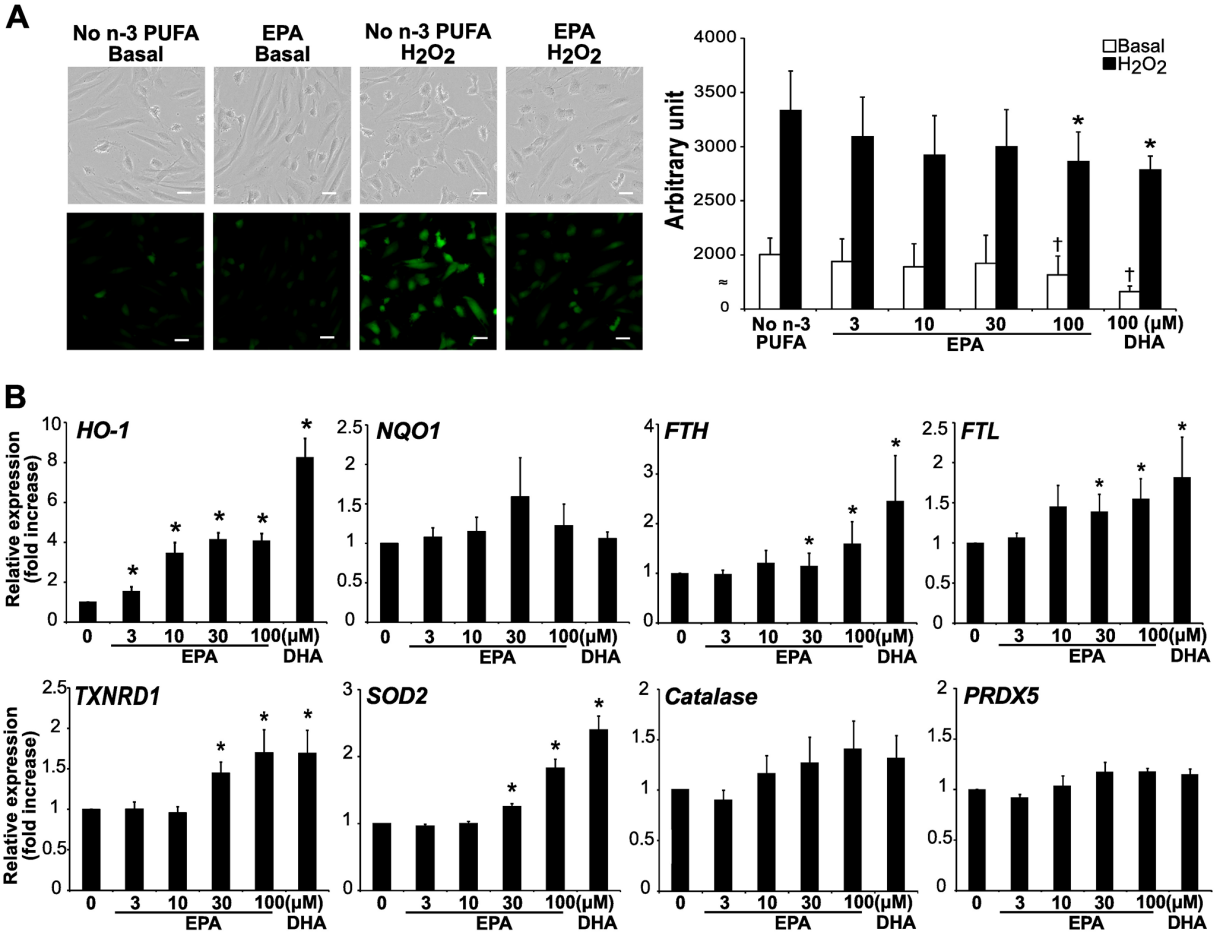
n-3 PUFAs reduce intracellular reactive oxygen species. (A) Intracellular ROS was detected by CM-H_2_DCFDA fluorescence in HAECs. Cells were incubated with H_2_O_2_ (100 μM) for 30 min after 24 h treatment with EPA (indicated doses) or DHA (100 μM). Representative images are shown in the left panels. Scale bar = 40 μm. Quantification of CM-H_2_DCFDA fluorescence is shown in the right panel. †*P*<0.05 compared with no n-3 PUFA/basal; *P<0.05 compared with no n-3 PUFA/H_2_O_2_ (n = 7). (B) Cells were treated with EPA (indicated doses) or DHA (100 μM) for 36 h. Total RNA was extracted and subjected to real-time RT-PCR analysis to quantitate the relative mRNA expression of antioxidant molecules. *P<0.05 compared with control (n = 9).

Since the decrease of H_2_O_2_-induced DSBs by n-3 PUFA treatment was not mediated through activation of DNA damage response, genome protective properties of n-3 PUFAs were further elucidated. Oxidative stress is a major source of DNA damage, thus we examined their effects on intracellular ROS in HAECs. Both EPA and DHA (100 μM) significantly reduced intracellular ROS under the basal condition (by 9.4% and 17.1%, respectively) and H_2_O_2_ stimulation (by 14.1% and 16.4%, respectively) (Fig 2A). We also examined mRNA levels of various anti-oxidative molecules. Treatment with EPA dose-dependently increased the levels of the mRNAs for HO-1, FTH, FTL, TXNRD1, and SOD2. Likewise, DHA (100 μM) significantly increased these molecules (Fig 2B). EPA and DHA did not affect the levels of the mRNAs for NQO1, catalase, PRDX5.

### *NRF2* silencing abrogates the n-3 PUFA-induced increases of antioxidant molecules

**Fig 3:**
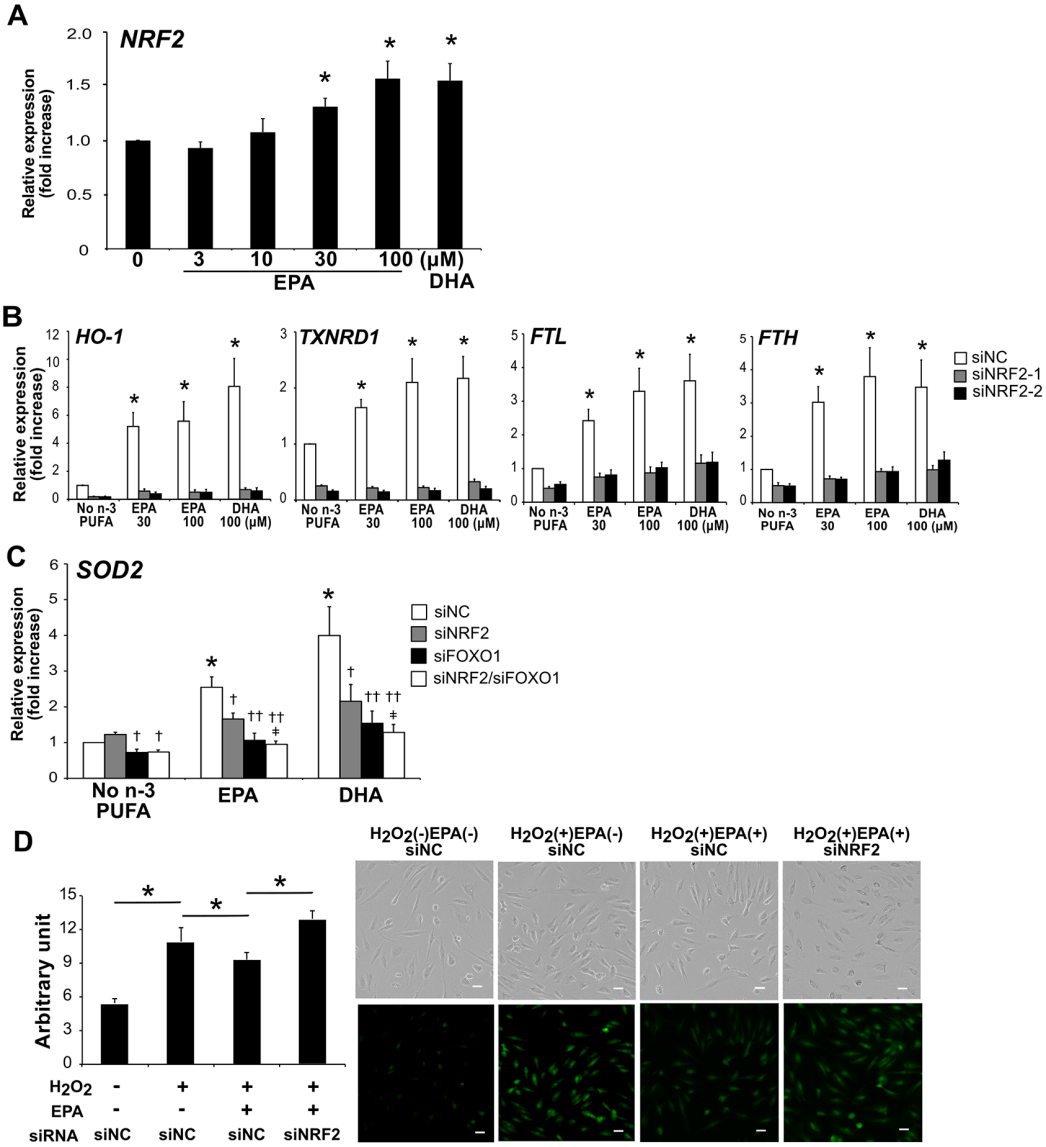
*NRF2* silencing abrogates the antioxidative effects of n-3 PUFAs. (A) Real-time RT-PCR analysis of *NRF2* in HAECs after 36 h treatment with EPA (indicated doses) or DHA (100 μM). *P<0.05 compared with control (n = 7). (B) Cells transfected with siRNA against *NRF2* (siNRF2-1 or siNRF2-2) and/or *FOXO1*, or negative control siRNA (siNC) were treated with EPA or DHA for 36 h. The mRNA expression of antioxidant molecules was quantified as described in (A). * *P*<0.05 compared with no n-3 PUFA (n = 5). (C) Cells were treated with EPA or DHA (100 μM) for 36 h. †*P*<0.05, ††*P*<0.01 compared with corresponding control; ‡*P*<0.05 compared with siNRF2 (n = 4). (D) Intracellular ROS was assessed by CM-H_2_DCFDA staining in *NRF2*-silenced HAECs. Quantification of CM-H_2_DCFDA fluorescence is shown in the left panel. *P<0.05 compared with indicated controls (n = 5). Representative images are shown in the right panels. Scale bar = 40 μm.

HO-1, FTH, FTL and TXNRD1 are all known to be regulated by the transcription factor nuclear factor erythroid 2-related factor 2 (NRF2), which is the master mediator of oxidative stress pathways [21]. Thus we examined whether the elevated levels of these antioxidant molecules by EPA and DHA were dependent on NRF2. HAECs were transfected either with two siRNA variants against *NRF2* (siNRF2-1 or siNRF2-2) or negative control siRNA (siNC). Transfection with NRF2 siRNAs almost completely abrogated the levels of the mRNAs for HO-1, TXNRD1, FTL and FTH induced by EPA and DHA (Fig 3B).

*SOD2* is one of the target genes of the forkhead box O (FOXO) family transcription factors [22]. So we silenced *FOXO1* in HAECs to examine whether the n-3 PUFA-induced increase of SOD2 mRNA level was FOXO dependent. As we expected, *FOXO1* knockdown abolished the n-3 PUFA-induced increase of SOD2 mRNA level. Interestingly, *NRF2* knockdown alone also abrogated it, and furthermore, double knockdown of *NRF2* and *FOXO1* enhanced this effect (Fig 3C). This effect was only seen in *SOD2* expression but not in the NRF2 target genes mentioned above (S2 Fig).

We also examined the effect of *NRF2* silencing on the reduction of intracellular ROS by n-3 PUFAs. Intracellular ROS was increased with H_2_O_2_, and reduced by EPA treatment, which was cancelled by RNA interference against *NRF2* (Fig 3D).

Additionally, we tested the effect of EPA on H_2_O_2_-induced gene expression associated with inflammation. mRNA levels of IL-6 and MCP-1 elevated by H_2_O_2_ stimulation were decreased with EPA treatment (Panel A in S3 Fig). Furthermore, this effect of EPA was cancelled by *NRF2* silencing (Panel B in S3 Fig).

### *NRF2* silencing cancels the anti-senescent effects of n-3 PUFAs

**Fig 4:**
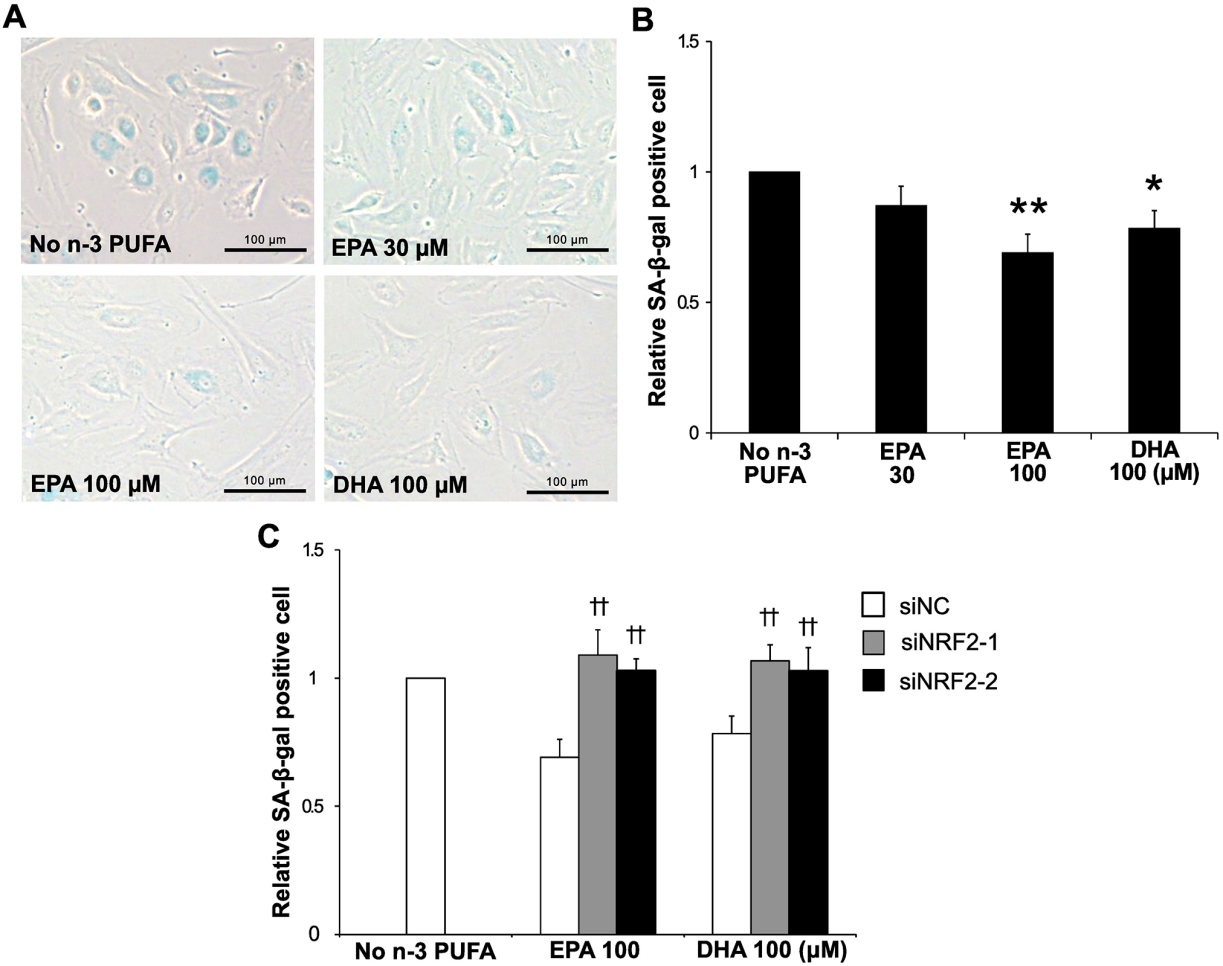
n-3 PUFAs reduce senescence-associated β-galactosidase activity. (A) Cells were treated with EPA (30 and 100 μM) or DHA (100 μM) for 36 h, and then subjected to SA-β-gal staining at 5 days following 1 h exposure to H_2_O_2_ (100 μM). (B) Quantification of relative SA-β-gal positive cells. *P<0.05, **P<0.01 compared with no n-3 PUFA (n = 6). (C) SA-β-gal activity in cells transfected with siRNAs against *NRF2* was assessed as described in (A). ††*P*<0.01 compared with corresponding control (n = 6).

One of the consequences of persistent DNA damage is cellular senescence [23]. Thus we examined the effect of EPA and DHA on H_2_O_2_-induced senescence in HAECs. As Fig 4A shows, EPA and DHA (100 μM) significantly reduced H_2_O_2_-induced SA-β-gal activity, a senescent marker (by 31% and 22%, respectively). Furthermore, this effect was revoked by *NRF2* silencing (Fig 4B).

## Discussion

Accumulating evidence suggests that DNA damage plays a pivotal role in the development of atherosclerosis [24, 25]. Although n-3 PUFAs likely possess heterogeneous properties beneficial to cardiovascular health, the effect of EPA and DHA on genome integrity in the context of atherosclerosis has been remained unknown [26]. One of the major findings of this study is that EPA and DHA diminished ROS-induced DNA damage in human aortic endothelial cells. H_2_O_2_-induced γ-H2AX foci formation was decreased by both EPA and DHA, which indicated that these n-3 PUFAs diminished DSBs, the most severe type of DNA damage. The decrease in DSBs by n-3 PUFAs was detected as early as 30 minutes after exposure of H_2_O_2_. H_2_O_2_-induced activation of ATM and DNA-PKcs, the key molecules that initiate DNA damage response, was also reduced by n-3 PUFA treatment. Additionally, the level of intracellular ROS was reduced by the treatment of EPA or DHA. These data suggest that the genome protective effects of these n-3 PUFAs were mediated by reducing inducers of DNA damage, ROS in this study, rather than promoting the DNA repair system.

ROS are constantly produced as by-products of normal cellular metabolism or from exposure to stimuli, such as ionizing radiation and chemicals. Reacting with DNA, ROS can cause various DNA lesions including abasic sites, oxidized bases, single-strand breaks and DSBs. Cells produce multiple ROS scavengers to defend themselves against such oxidative threats [21, 27]. We observed the increased mRNA levels of antioxidant molecules by n-3 PUFAs. HO-1 degrades heme and generates biliverdin, carbon oxide and iron. Biliverdin is converted to bilirubin by biliverdin reductase, and both of these bile pigments efficiently scavenge peroxyl radicals [28, 29]. Our data also showed that n-3 PUFAs increased the levels of the mRNAs for FTH and FTL, which are components of ferritin complex. FTH has a ferroxidase activity that converts Fe(II) into Fe(III), whereas FTL sequestrates Fe(II), together eliminates Fenton reaction, which produces highly reactive hydroxyl radicals using Fe(II) [30]. TXNRD1 reduces, and thereby, regenerates thioredoxin, a potent anti-oxidant molecule, using NADPH [31]. These data indicate that n-3 PUFAs induce the expression of these antioxidants, reduce ROS, and as a result, diminish DNA damage of the cell.

Our data demonstrated n-3 PUFAs increased the level of the mRNA for NRF2, a master transcriptional regulator of response to ROS. Additionally, knockdown of *NRF2* markedly blunted the n-3 PUFA-induced increases of HO-1, TXNRD1, FTH, and FTL mRNAs. Compatible with these data, EPA reduced intracellular ROS and this effect was also cancelled by NRF2 silencing. Kusunoki *et al*. reported that n-3 PUFAs induce antioxidant response by mediating Nrf2/HO-1 pathway in 3T3 adipocytes, and in later study, 4-hydroxy hexenal, a derivative of DHA, improves human endothelial function via NRF2 activation [32, 33]. Our data are consistent with them in which EPA and DHA did not affect the mRNA expression of catalase and glutathione peroxidase. In their study neither EPA nor DHA affected the mRNA expression of NRF2 and SOD2, but we observed the increased mRNA expression of these molecules (Figs 2B and 3A). This may be due to difference in cells, that is, they used endothelial cells originated in umbilical vein, ours in aorta. n-3 PUFA-induced antioxidant responses are slightly different depending on the cells. Gao *et al*. have reported n-3 PUFA oxidation products dissociate the binding of Kelch-like ECH-associated protein 1 (Keap1) to NRF2, stabilize, and thereby activating NRF2-mediated antioxidant response [34]. It is likely that both transcriptional regulation and Keap1-regulated stabilization of NRF2 are involved in n-3 PUFA-induced expression of these antioxidants. Our data demonstrated that several antioxidants other than HO-1 are upregulated by n-3 PUFAs for the first time.

Our data also showed that the level of the mRNA for SOD2, a mitochondrial manganese SOD, was significantly increased by EPA and DHA. Unlike other antioxidants we evaluated, the n-3 PUFA-induced expression of SOD2 mRNA was abrogated by *NRF2* silencing only modestly, and also significantly abrogated by *FOXO1* silencing. In addition, a combination of the two silencing had a slightly but significantly additive effect. FOXOs are highly conserved transcription factors which regulate various cellular stress responses including metabolic stress and oxidative stress [35]. Collectively, these results suggest that NRF2 and FOXO1 may have cooperatively regulated the SOD2 mRNA level induced by n-3 PUFA. Interestingly, *NRF2* silencing drastically abrogated n-3 PUFA-induced mRNA levels of HO-1, TXNRD1, FTH and FTL, yet we observed slight increases of these genes by EPA and DHA (Fig 3B). Although we could not find significant effects of *FOXO1* silencing on n-3 PUFA-induced expression of these NRF2-regulated antioxidants, it is possible that transcriptional factors other than NRF2, such as another FOXO family members, may also be involved. Further studies are necessary to clarify the transcriptional mechanisms for n-3 PUFA-induced expression of antioxidants.

Sustained DNA damage results in cellular senescence, apoptosis and inflammation. Lee *et al*. recently reported that EPA had protective effects on H_2_O_2_-induced cell death [36]. The present study demonstrated that n-3 PUFAs attenuate oxidative stress-induced inflammatory gene expression (S3 Fig) and senescence (Fig 4) in a NRF2-dependent manner. Interestingly, prospective cohort study by Farzaneh-Far et al. demonstrated inverse relationship between blood levels of marine n-3 PUFAs at baseline and the rate of telomere shortening over 5 years in patients with coronary artery disease [37]. ROS specifically target a certain sequence in telomere and causes telomeric attrition, a sort of DNA damage. n-3 PUFAs may decelerate cell senescence partly by inhibiting ROS-induced telomere attrition. In addition, using transgenic mice, Gray *et al*. demonstrated that ineffective DSB repair modifies plaque phenotype and may contribute to vulnerable plaques [38]. Taking these into account, n-3 PUFAs may prevent atherosclerotic plaque progression and promote plaque stability partly by inhibiting DNA damage and subsequent cell senescence.

The difference in the cardiovascular benefits of EPA and DHA has been remained unclear. Recent clinical studies suggest that EPA, but not DHA, seems to efficiently improve lipid profiles and reduce inflammatory biomarkers in patients with CVD risk factors [39, 40]. In addition, a clinical study conducted by Iwamatsu *et al*. suggests that EPA may be more beneficial than DHA for preventing acute coronary disease in patients with coronary artery disease [41]. Since EPA, compared to DHA, seems to provide more benefits on CVD, we put more focus on the effect of EPA in this study. Our data showed, at least in HAECs, that EPA and DHA seem to have similar roles for genome protection such as attenuation of H_2_O_2_-induced DNA damage and upregulation of the mRNA expression of antioxidant molecules via NRF2.

In conclusion, our findings indicate that n-3 PUFAs attenuate oxidative DNA damage and subsequent cell senescence, at least in part, through upregulation of NRF2-mediated antioxidant response. Therefore mitigating DNA damage may be a possible mechanism by which n-3 PUFAs exert cardioprotective effects. Although it is necessary to further elucidate the detailed molecular action of n-3 PUFAs on cardiovascular health, our findings support n-3 PUFAs as potential therapeutic agents against CVD.

## Supporting information

S1 Fig Effect of n-3 PUFAs on H_2_O_2_-induced DNA-PKcs activation. Cells were treated with H_2_O_2_ (100 μM) for 1 h after treatment with EPA or DHA (indicated doses) for 48 h. Whole cell lysates were subjected to western blot analysis of phosphorylated DNA-PKcs (S2056).
(SVG)

S2 Fig *FOXO1* silencing does not affect n-3 PUFA-induced expression of antioxidant molecules in HAECs. Cells were transfected with siRNA against *NRF2* and/or *FOXO1*, or negative control siRNA (siNC) and then treated with EPA or DHA (100 μM) for 36 h. Relative mRNA expression of *HO-1*, *TXNRD1*, *FTL*, *FTH* to 18s ribosomal RNA was quantitated using real-time RT-PCR (n = 8).
(SVG)

S3 Fig Effect of *NRF2* silencing on H_2_O_2_-induced inflammatory gene expression. (A) Cells treated with or without EPA (100 μM) for 36 h were exposed to H_2_O_2_ (100 μM) for 1 h. Total RNA was extracted and subjected to real-time RT-PCR analysis to assess the mRNA levels of *IL-6* and *MCP-1*. (B) Cells were transfected either with negative control siRNA (siNC) or siRNA against *NRF2* and then treated with EPA (100 μM) for 36 h. All samples were exposed to H_2_O_2_ (100 μM) for 1 h. Data were analyzed by Student’s *t*-test. *P<0.05 and **P<0.01 (n = 4 for each).
(SVG)

S1 Table Sequences of the primers used in the real-time RT-PCR analysis. (DOCX)
