# Supplementary material for: Fish oil omega-3 polyunsaturated fatty acids attenuate oxidative stress-induced DNA damage in vascular endothelial cells
Source: PLoS One. 2017 Nov 9;12(11):e0187934. doi: 10.1371/journal.pone.0187934 (PMC5679535; doi:10.1371/journal.pone.0187934)
Supplement: S1 Table — (DOCX) [file pone.0187934.s004.docx]

**S1 Table. Sequences of the primers used in the real-time PCR analysis**.

| Name | 5’-3’ sequence | |
| --- | --- | --- |
|  | Forward primer | Reverse primer |
| *HO-1* | CAGGAGCTGCTGACCCATGA | AGCAACTGTCGCCACCAGAA |
| *NQO1* | GAGTCGGACCTCTATGCCATGAA | AGAACAGACTCGGCAGGATACTGAA |
| *FTH* | GCCAGAACTACCACCAGGACTCA | TGGTTCTGCAGCTTCATCAGTTTC |
| *FTL* | AGCCAGCTGAAGATGAGTGG | TGAGTCTCCAGGAAGTCACAGA |
| *TXNRD1* | ACTTGGGCATCCCTGGTGAC | GCGCACTCCAAAGCGACATA |
| *SOD2* | CGGCCTACGTGAACAACCTG | GCTATGTTGATATGACCACCACCA |
| *Catalase* | GGTCATGACATTTAATCAGGCAGAA | TTGCTTGGGTCGAAGGCTATC |
| *PRDX5* | GATTCGCTGGTGTCCATCTTTG | AGGCCTGTGCCATCTGGTTC |
| *NRF2* | AGCCCAGCACATCCAGTCAG | TGCATGCAGTCATCAAAGTACAAAG |
| *IL-6* | AAGCCAGAGCTGTGCAGATGAGTA | TGTCCTGCAGCCACTGGTTC |
| *MCP-1* | GCTCATAGCAGCCACCTTCATTC | GGACACTTGCTGCTGGTGATTC |
| 18s rRNA | ACTCAACACGGGAAACCTCA | AACCAGACAAATCGCTCCAC |
